# Metabolomic and inflammatory signatures in congenital hypothyroidism: a longitudinal analysis of levothyroxine response

**DOI:** 10.3389/fendo.2026.1824072

**Published:** 2026-07-01

**Authors:** Marcela Vela-Amieva, Isabel Ibarra-González, Raúl Calzada León, María de la Luz Ruiz-Reyes, María Eugenia Constantini, Sara Guillén-López, Lizbeth López-Mejía, Michelle Citlalli Luna-Nequiz, Rosa Itzel Carrillo-Nieto, Cynthia Fernández-Lainez

**Affiliations:** 1 Laboratorio de Errores Innatos del Metabolismo y Tamiz, Instituto Nacional de Pediatría, Secretaría de Salud, Ciudad de México, Mexico; 2 Instituto de Investigaciones Biomédicas, Universidad Nacional Autónoma de México (UNAM), Ciudad de México, Mexico; 3 Departamento de Endocrinología, Instituto Nacional de PediatríaSecretaría de Salud, Ciudad de México, Mexico; 4 Laboratorio de Hormonas, Instituto Nacional de Pediatría, Secretaría de Salud, Ciudad de México, Mexico; 5 Facultad de Ciencias, Universidad Nacional Autónoma de México, UniversidadNacional Autónoma de México (UNAM), Ciudad de México, Mexico

**Keywords:** biomarkers, birth defects, congenital hypothyroidism, metabolomics, precision medicine, thyroid

## Abstract

**Introduction:**

Congenital hypothyroidism (CH) requires early levothyroxine (LT4) replacement to prevent neurodevelopmental impairment. Treatment monitoring relies primarily on thyroid-stimulating hormone (TSH) and thyroxine (T4); however, these markers may not fully capture systemic metabolic changes during therapy. We aimed to explore metabolomic and inflammatory signatures associated with the biochemical response to LT4 treatment in pediatric patients with CH.

**Methods:**

A prospective longitudinal study was conducted in 11 pediatric CH patients. Plasma samples were collected at diagnosis (Pre Tx) and after biochemical euthyroidism was achieved (Post Tx; mean follow-up 2.7 months). Metabolomic profiling was performed using tandem mass spectrometry. Inflammatory status was assessed by measuring plasma tumor necrosis factor-alpha (TNF-α) and interleukin 10 (IL-10). Nutritional status was also evaluated.

**Results:**

A group of nine metabolites discriminated between Pre Tx and Post Tx samples. The main metabolic changes involved sphingolipid metabolism, characterized by reduced ceramides (Cer) and hexosylceramides (HexCer), together with increased levels of specific sphingomyelins (SM). Circulating TNF-α and IL-10 concentrations were markedly elevated at diagnosis and remained elevated after treatment. Although both cytokines showed a decreasing trend following LT4 therapy, no statistically significant differences were observed between time points. Nutritional assessment showed a modest increase in length-for-age Z-score in the Post Tx group. Given the recognized roles of sphingolipids in cellular signaling and inflammatory regulation, this lipid changes may reflect broader metabolic adaptations during treatment.

**Conclusions:**

LT4 therapy in CH pediatric patients was associated with changes in circulating sphingolipids, suggesting remodeling of sphingolipid metabolism after treatment initiation. Metabolomic profiling may provide complementary information on metabolic changes occurring during therapy and could contribute to a more detailed characterization of treatment response in CH.

## 1 Introduction

Congenital hypothyroidism (CH) is the most common endocrine disorder in the neonatal period, with an estimated birth prevalence of 1 in 2,000 to 3,000 live births. However, this rate varies by ethnicity, with Asian and Hispanic populations being more frequently affected (1). CH is characterized by insufficient production or action of thyroid hormones, which are critical for early neurodevelopment, myelination (2), and metabolic homeostasis (3). If left untreated, CH leads to profound and irreversible neurological impairment, including severe intellectual disability, motor dysfunction, and growth retardation. These adverse outcomes underscore the crucial role of thyroid hormones in fetal and early postnatal development, particularly in the nervous system (4).

In recent years, advances in precision medicine and high-throughput technologies have enabled a more comprehensive understanding of the systemic effects of CH beyond its classical clinical presentation.

Metabolomic profiling has emerged as a powerful tool for exploring systemic metabolic alterations in thyroid dysfunction, particularly in CH (5, 6). In this context, neonatal screening using dried blood spots has revealed significant disturbances in amino acid turnover, lipid, and acylcarnitine profiles, suggesting early metabolic dysregulation in CH patients (7, 8). Notably, altered beta-oxidation of long-chain fatty acids and relative changes in linoleoylcarnitine levels indicate disease-specific disruptions in lipid catabolism during the neonatal period in CH patients (8). Similar metabolic alterations have been observed in acquired hypothyroidism, where decreased levels of branched-chain amino acids and disrupted lipid profiles reflect the systemic impact of thyroid hormone deficiency (9, 10).

Lipidomic studies have further deepened the understanding of these alterations. Blazewicz et al. demonstrated significant changes in plasma lysophosphatidylcholines and phosphatidylcholines in individuals with hypothyroidism, and these changes persisted despite normalization of thyroid hormone levels with levothyroxine treatment, highlighting the limitations of conventional endocrine biomarkers in capturing subtle metabolic dysfunctions (11). Beyond metabolism, recent studies have revealed a potential link between thyroid disorders and immune-inflammatory responses. While overt inflammation is not a classic characteristic of CH, evidence suggests low-grade chronic inflammation and oxidative stress in hypothyroid patients (7, 12, 13). Thyroid hormones are increasingly recognized for their modulatory effects on immune cells and cytokine production, interacting with pathways such as the inflammasome and Wnt/β-catenin signaling (14). On the other hand, thyroid hormones may affect the antioxidant balance, since thyroid dysfunction has been associated with oxidative stress in animals and humans (15, 16).

Nevertheless, the intricate crosstalk between thyroid function, lipid and amino acid metabolism, and immune regulation remains incompletely understood.

This study aimed to evaluate metabolic and inflammatory changes before and after thyroid hormone replacement therapy in pediatric patients with confirmed CH, using metabolomic and inflammatory biomarkers, to identify disease signatures.

## 2 Materials and methods

### 2.1 Studied population and design

A prospective observational cohort study was conducted. The study was conducted at the National Institute of Pediatrics and included pediatric patients with confirmed CH. A case was considered confirmed when an abnormal thyroid profile consistent with CH was documented. Thyroid scintigraphy with technetium-99m pertechnetate was performed to assess the presence, location, and uptake of the thyroid. Anterior neck images were acquired using a gamma camera, allowing etiological differentiation between thyroid dysgenesis and dyshormonogenesis. A neck ultrasound was also performed to assess the presence, absence, and characteristics of the thyroid gland when scintigraphy did not show thyroid gland uptake. Knee bone age measurements were performed in all patients to classify them as having CH of intrauterine or extrauterine onset.

### 2.2 Patient evaluation

We recruited 11 newly diagnosed, untreated patients with CH. Two sequential samples were obtained from each patient:

Group 1: Pretreatment (Pre Tx). Samples were collected at presentation to the reference center, prior to initiation of levothyroxine therapy. Levothyroxine was administered at a standardized dose of 10-15 µg/kg/day.
Group 2: Posttreatment (Post Tx). Samples were collected after achieving biochemical euthyroidism, operationally defined as serum TSH and free T4 concentrations within the age-appropriate reference ranges.

### 2.3 Sample collection

Two mL of venous blood were collected at both study time points (Pre Tx and Post Tx). Blood samples were centrifuged to obtain serum. Thyroid profile analysis was performed immediately, and the remaining sample was stored at -70 °C until the metabolomic analysis.

### 2.4 Thyroid profile

Serum thyroid hormone levels were measured before and after treatment at the Biochemistry and Endocrinology Laboratory of the National Institute of Pediatrics using chemiluminescent immunoassays (Immulite 2000 XPi, Siemens, USA), according to the manufacturer´s instructions. TSH was determined by solid-phase, two-site chemiluminescent immunometric assay (calibration range 0.004–75 IU/mL; imprecision <6.6%). Total T3 and total T4 were measured using solid-phase competitive chemiluminescent enzyme immunoassays (T3: 40–600 ng/dL, imprecision <9.0%; T4: 1.0-24 μg/dL, imprecision <8.0%). Free T3 and free T4 were assessed by competitive chemiluminescent immunoassays (FT: 1.0–40 pg/mL, imprecision <8.0%; FT4: 0.3-6.0 ng/dL, imprecision <7.0%). Analytical performance was validated using the internal quality control program Acusera 24/7 and the External Quality Assessment scheme RIQAS (Randox, Ireland).

### 2.5 Nutritional analysis

The general nutritional status of patients with CH was evaluated in both studied groups (Pre Tx and Post Tx). To that end, weight and height were measured using a digital Seca scale, an infantometer, and a stadiometer (Hamburg, Germany), and the BMI Z-score was also obtained. Patients were measured with minimal clothing. For patients aged 0 to 5 years, BMI Z-score was calculated using Anthro (Version 3.2.2.1, Geneva, Switzerland), and for patients aged 5 to 19 years, Anthro Plus (Version 1.0.4, Geneva, Switzerland) was used. BMI Z-score was classified based on the WHO’s criteria: For children younger than 5 years, a BMI Z-score of -2 or less was defined as underweight, normal weight as -1.99 to 0.99, at risk of overweight; + 1 to + 2, overweight: + 2 to + 3, and + 3 or more obesity. For patients older than 5 years, underweight was defined as < −2 SD, normal weight between − 2 and + 1 SD, overweight > + 1 SD, and obesity > + 2 SD. Stunting was defined as a height-for-age Z-score < - 2 SD (17). The results are presented in box-and-whisker plots, of the median with 5–95 quartiles.

### 2.6 Metabolomic analysis

To investigate differences in the metabolome of CH patients before and after treatment initiation, a targeted metabolomic analysis was performed. The assay identified and quantified 645 endogenous metabolites, including amino acids and amino acid derivatives, biogenic amines, ceramides, cholesterol esters, diacylglycerols, acylcarnitines, glycerophospholipids, sphingomyelins, triacylglycerols, organic acids, and nucleotides/nucleosides. A targeted quantitative metabolomics approach (TMIC MEGA™) was employed to analyze plasma samples using a combination of direct flow injection mass spectrometry (DFI-MS/MS) and liquid chromatography tandem mass spectrometry (LC-MS/MS). This high-throughput method enables the identification and quantification of up to 645 endogenous metabolites from 40 μL of plasma, including amino acids and their derivatives, biogenic amines, ceramides, cholesterol esters, acylcarnitines, diacylglycerols, glycerophospholipids, sphingomyelins, triacylglycerols, organic acids, and nucleotides/nucleosides.

Sample preparation involved thawing plasma on ice, vortexing, and centrifugation, followed by derivatization using phenylisothiocyanate (PITC) for amino acids, biogenic amines, and related metabolites. No derivatization was required for lipid and acylcarnitine analysis via DFI-MS/MS. Organic acids were derivatized using 3-nitrophenylhydrazine (3-NPH) following protein precipitation with cold methanol.

Quantification was achieved using isotope-labeled internal standards (ISTDs) and external calibration curves spanning physiological and pathological concentration ranges. Up to 92 analytical standards and 48 ISTDs were used for amino acids and nucleotide/nucleoside analyses, while organic acids were quantified using up to 107 standards and matched isotope-labeled derivatives. Extracted analytes were analyzed using an AB Sciex 5500 QTrap^®^ MS coupled to an Agilent 1290 UHPLC system, operated in multiple reaction monitoring (MRM) mode. Chromatographic separation was performed using a reverse-phase C18 column for LC-MS/MS assays. DFI-MS/MS was applied to lipid-related metabolites using a solvent-based flow program without chromatographic separation. Data acquisition and quantification were performed using Analyst 1.7.2 and MultiQuant 3.0.3 software, with peak integration and quality control processing facilitated by in-house profiling software. The metabolomic analysis was performed using MetaboAnalyst 6.0 web platform (https://dev.metaboanalyst.ca).

### 2.7 Cytokine quantification

To investigate the inflammatory state of CH patients before and after the levothyroxine treatment, the secretion of the anti-inflammatory cytokine interleukin 10 (IL-10) and the proinflammatory cytokine tumor necrosis factor-alpha (TNFα) was quantified in the patients’ plasma by a colorimetric ELISA assay (R&D SYSTEM, Minneapolis, MN, USA) according to the manufacturer’s protocol. To determine the TNFα and IL-10 reference values, these cytokines were measured in plasma samples from five healthy controls using the same methodology used to quantify the patients’ samples.

### 2.8 Statistical analysis

To assure the quality and reliability of the metabolomic analysis, only metabolites that met stringent statistical criteria were considered for further evaluation. In this sense, only metabolites with fewer than 20% missing values were retained for analysis (18). To generate appropriate Gaussian distributions, the data were normalized by sum, cube root transformed, and auto-scaled. We used multivariate and univariate analysis. To compare metabolite data across and between the studied groups, we used partial least squares discriminant analysis (PLS-DA) score plots. The performance of the PLS-DA model was evaluated using the cumulative R2Y and Q2 values. Specifically, R2Y was used to assess goodness of fit, while Q2 served as an indicator of the model’s predictive capacity via cross-validation. Additionally, only metabolites with variable importance in projection (VIP) scores > 2.0 were included in the analyses, as these indicate a strong contribution to the separation among groups. To prevent overfitting and determine the optimal number of classification components, a 5-fold cross-validation (5-fold CV) approach was implemented using the MetaboAnalyst 6.0 web platform. A maximum of 5 components were searched, and the Q2 metric was utilized as the primary performance measure to select the final model. Data were analyzed using PLS-DA and univariate methods with stringent selection criteria: Variable Importance in Projection (VIP) > 2.0, Fold Change (FC) > 2, and FDR-corrected p-value < 0.05 (19, 20). This conservative approach was adopted to identify the most robust metabolic drivers of the response to levothyroxine and to minimize the risk of false-positive discoveries inherent to high-dimensional metabolomic datasets. Univariate analysis for the identification of differential metabolites was performed using a Volcano plot within the MetaboAnalyst 6.0 web platform. The fold change (FC) was calculated as the ratio of Pre Tx to Post Tx analyte concentrations. Metabolites were considered significantly altered if they simultaneously met an FC threshold of > 2.0 or < 0.5, reflecting a significant decrease or increase Post Tx, respectively, and a p-value adjusted by the false discovery rate (FDR, Benjamini-Hochberg method) of 0.05. Measures of central tendency and dispersion were used for continuous data. The data distribution was assessed using the Shapiro-Wilk test. For data with a parametric distribution, one-way ANOVA, mixed-effects analysis with Geisser-Greenhouse correction, and Dunnett’s multiple comparison tests were performed, and the results were presented as means ± SDs. When data were nonparametrically distributed, the Wilcoxon matched pairs signed rank test was performed. A p-value < 0.05 was considered statistically significant (*p < 0.05, **p < 0.01, ***p < 0.001, ****p < 0.0001).

## 3 Results

### 3.1 Clinical characteristics of patients and thyroid profiles

A total of 11 patients were included in the study, comprising six females and five males. Their clinical and demographic characteristics are summarized in **Supplementary Table 1**. Regarding the type of CH, five patients (45.45%) were presented with thyroid agenesis, five (45.45%) with sublingual ectopic thyroid tissue, and one (9.1%) with thyroid hypoplasia.

**Figure 1:**
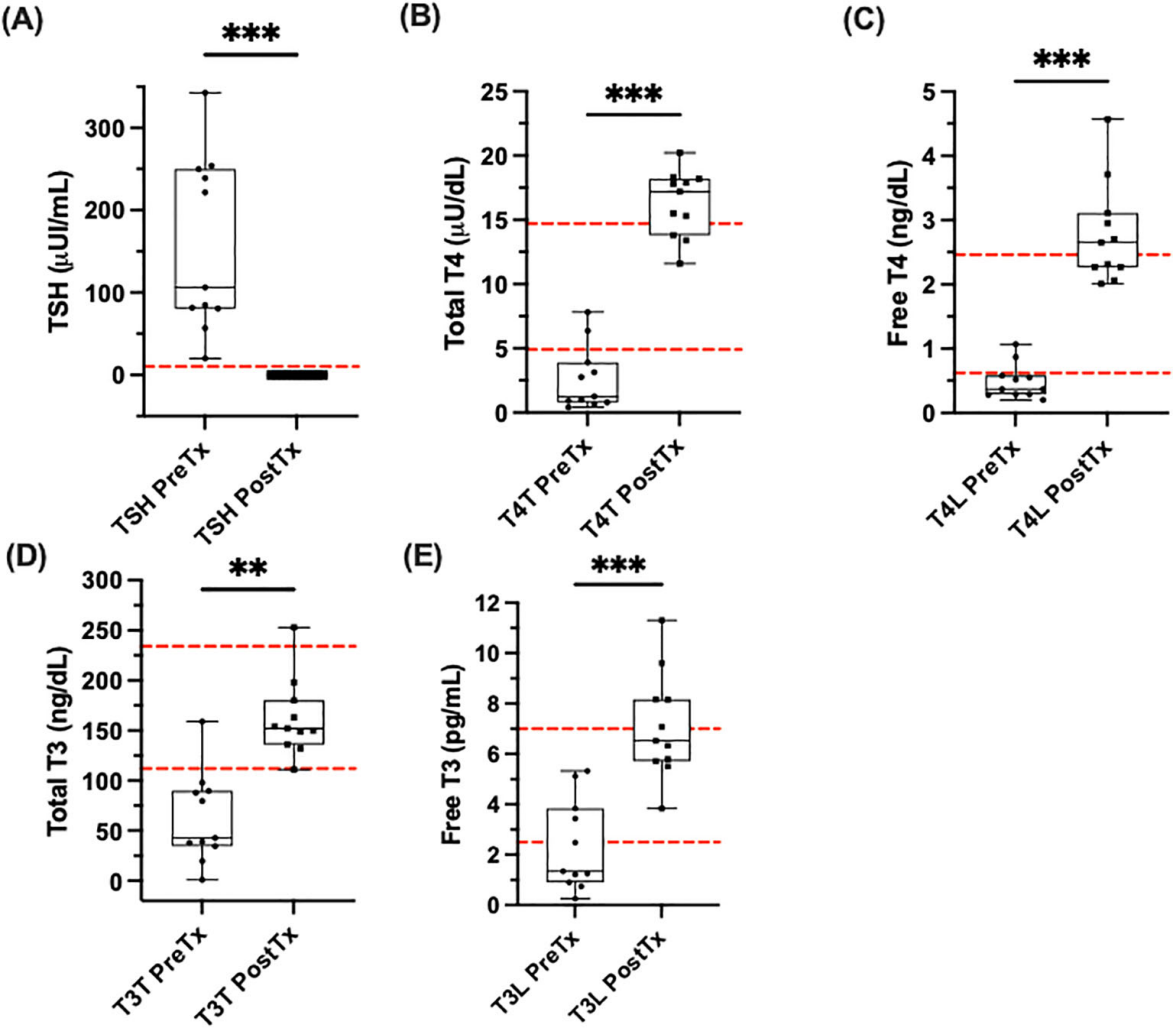
. Box and whiskers plot of plasma thyroid profile of patients with congenital hypothyroidism before and after treatment with levothyroxine. Laboratory reference values in red dotted lines: **(A)** TSH (μUI/mL); **(B)** Total T4 4.9-14.7 (μU/dL); **(C)** Free T4 0.62-2.46 (ng/dL); **(D)** Total T3 112-234 (ng/dL); **(E)** Free T3 2.5-7 (pg/mL). Median (line in the box) with 5-95 quartiles of the thyroid hormones is presented. To investigate statistical differences between both studied groups, Wilcoxon matched pairs signed rank test was performed. ** p < 0.01; *** p < 0.001.

Box and whiskers plot of the thyroid profiles of the patients before and after treatment initiation are shown in **Figure 1**. The median TSH concentration was 106.1 UI/mL in patients before treatment initiation. After treatment, this value significantly decreased to normal levels, with a median of 0.03 UI/mL (**Figure 1A**). This change to the euthyroid state was achieved in a mean of 2.7 months (range 1–6 months). Overall, the thyroid profile (Total T4, free T4, total T3, and free T3) returned to normal concentrations in all patients after initiation of levothyroxine (**Figures 1B–E**).

### 3.2 Nutritional status results

**Figure 2:**
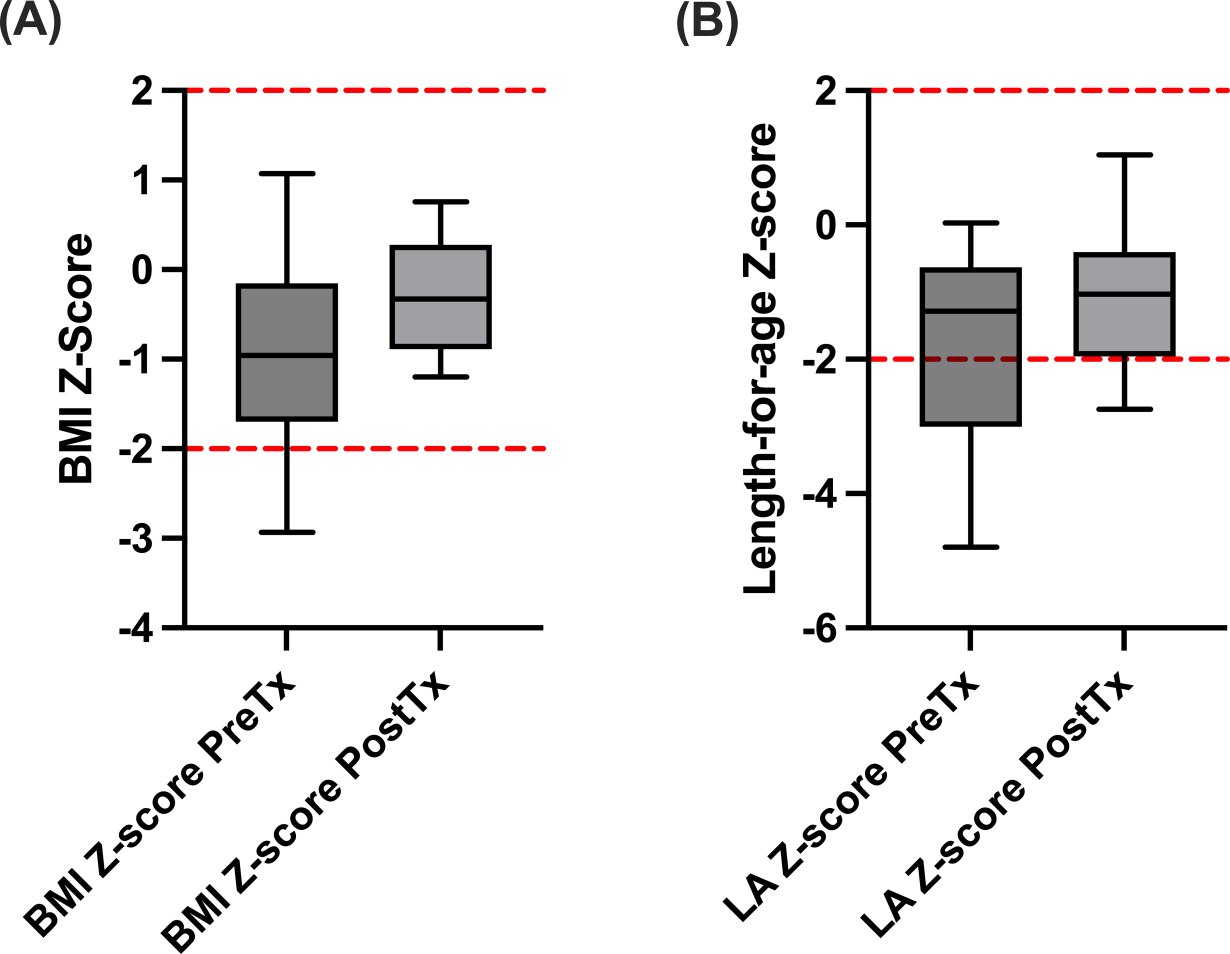
Box and whiskers plot of nutritional status of patients with congenital hypothyroidism before and after the initiation of levothyroxine treatment. **(A)** Z-score for body mass index. **(B)** length-for-age Z-score. To investigate statistical differences between both studied groups, Wilcoxon matched pairs signed rank test was performed. The dotted lines represent the normal Z-score cut-offs (-2 and +2).

**Table 1** shows the nutritional status of the CH patients. **Figure 2** presents the box and whiskers plot of the Z-scores for body mass index and length-for-age in the CH patients, before and after thyroid hormone replacement treatment, as measured by standardized anthropometric indicators. Panel A shows the Z-score for body mass index (BMI). Each box represents the BMI Z-score for the patients before and after treatment. A wide interindividual variation was observed both before and after treatment. For most patients, BMI Z-scores remained within a similar range after treatment, although some showed mild increases or decreases. No consistent directional trend was observed across the cohort, suggesting heterogeneous responses in weight status following hormone replacement. Panel B displays the length-for-age Z-scores, again before and after thyroid hormone replacement. Like BMI, individual responses varied. Some patients showed a slight increase in length-for-age Z scores after treatment, possibly reflecting compensatory growth. Overall, changes in this parameter appeared modest, and no clear pattern of improvement or decline was observed in the studied groups.

**Table 1:** Nutritional status of the patients, pre- and post-treatment, and according to the congenital hypothyroidism type.

Type of CH by gamma scan study	Patient ID	Sex	Nutritional diagnosis pre Tx	Nutritional diagnosis post Tx
Agenesis	2	F	Eutrophic	Eutrophic
	5	M	Eutrophic	Eutrophic
	7	F	Stunted	Eutrophic
	8	F	Eutrophic	Eutrophic
	9	F	Stunted/underweight	Stunted
	10	M	Eutrophic	Eutrophic
Sublingual node	1	M	Stunted	Eutrophic
	3	F	Stunted/obese	Stunted/overweight
	4	M	Eutrophic	Eutrophic
	6	F	Stunted/underweight	Stunted
	11	M	Eutrophic	Eutrophic

### 3.3 Metabolomics results

**Figure 3:**
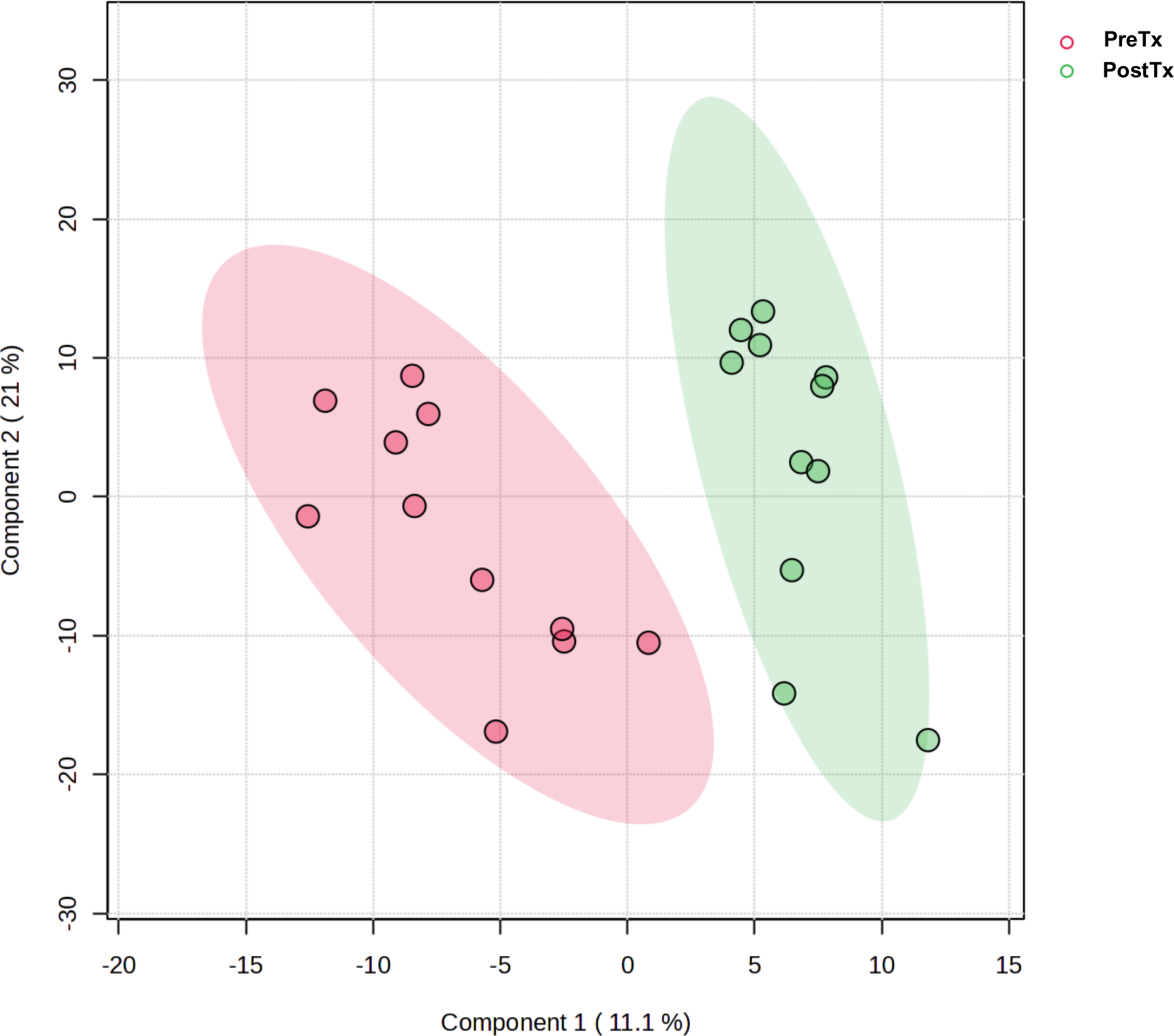
Partial least squares-discriminant analysis (PLS-DA) plot of differential metabolites in patients with congenital hypothyroidism pre- and post- the onset of treatment. The ellipse represents the 95% confidence interval based on Hotelling’s T2 statistics. Score plot derived from the PLS-DA model demonstrating group separation. Model robustness is indicated by R2Y = 0.9 (goodness-of-fit) and Q2 = 0.6 (predictive capacity). Q2 was cross-validated to ensure model stability. The optimal number of classification components was determined via 5-fold cross-validation (5-fold CV), with model performance evaluated based on the Q2 score.

PLS-DA was performed to evaluate global differences in the multivariate profiles of subjects before (Pre Tx) and after treatment (Post Tx). The PLS-DA score plot shows a clear separation between the Pre Tx (red) and Post Tx (green) groups along the first two principal components (Component 1: 11.1%, Component 2: 21.0%). Each point represents an individual sample, and the 95% confidence ellipses for each group indicate distinct clustering patterns. This separation suggests that the treatment induced substantial changes in the overall profile analyzed (**Figure 3**).

**Figure 4:**
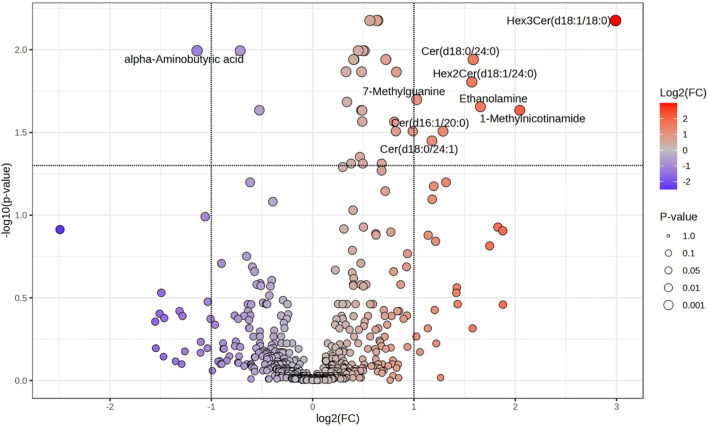
Volcano map highlighting those metabolites with a Variable Importance in Projection >2. False Discovery Rate < 0.05, and Fold Change threshold >2 of the metabolites analyzed from congenital hypothyroidism patients before and after the treatment onset. Wilcoxon matched pairs signed rank test was performed. Statistical significance [-log_10_ or false discovery rate (FDR)-adjusted p-value] on the Y-axis is plotted against the magnitude of change [log_2_ Fold Change (FC)] on the X-axis. The FC was calculated as the Pre Tx/Post Tx ratio. Consequently, positive log_2_ FC values (right side, red dots) represent metabolites that are significantly elevated Pre Tx and thus decrease following the treatment. Conversely, the negative log_2_ FC value (left side, blue dots) represents a metabolite that increases after treatment. Gray dots represent compounds that did not meet both selection criteria.

A volcano plot was used to visualize the differential features between Pre Tx and Post Tx groups. Each point represents an individual feature, with the X-axis corresponding to the log_2_ fold change (log_2_FC) and the Y-axis corresponding to the -log_10_ adjusted p-value (FDR-corrected). Features that were significantly higher in the Post Tx group (log_2_FC >1, FDR < 0.05) are highlighted in red, while those significantly lower (log_2_FC<-1, FDR 0.05) are shown in blue. The remaining non-significant features are depicted in gray. This analysis revealed a subset of features that were significantly altered following treatment, indicating a robust biological response (**Figure 4**).

**Figure 5:**
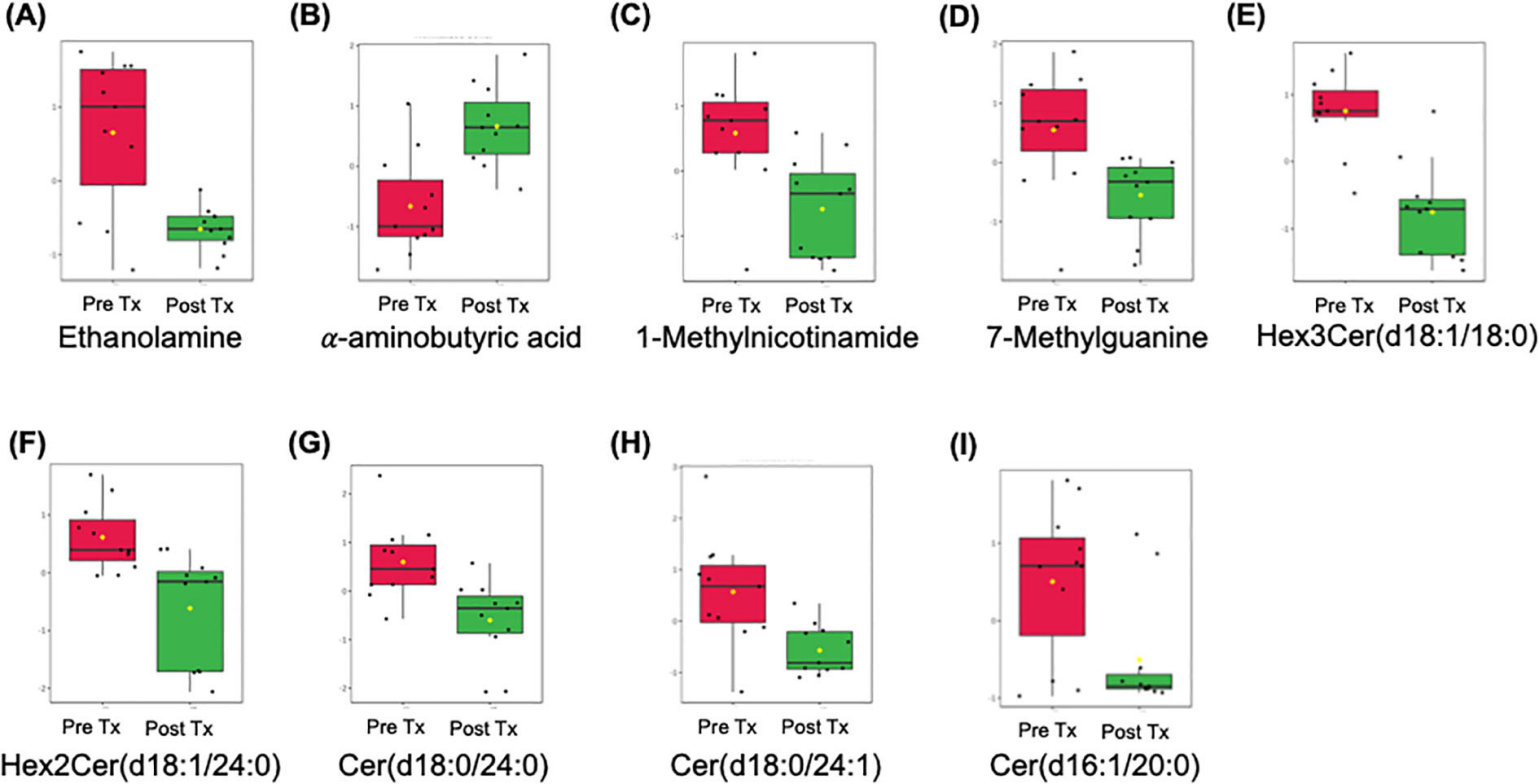
Box and whiskers plot of normalized serum concentrations of the nine metabolites **(A–I)** with Variable Importance in Projection >2, False Discovery Rate < 0.05, and Fold Change threshold >2 in patients with congenital hypothyroidism before and after treatment onset (n = 11). To investigate statistical differences between both studied groups, Wilcoxon matched pairs signed rank test was performed.

In **Figure 5**, box and whiskers plots illustrate the relative abundance of nine (9/642, 1.4%) metabolites that showed statistically significant differences between untreated CH patients (Pre Tx, red), and those treated with levothyroxine (Post Tx, green). The Pre Tx group was characterized by a distinct metabolic profile, with elevated concentration of several metabolites, including ethanolamine, 1-Methylnicotinamide, 7-Methylguanine, Hex3Cer(d18:1/18:0, Hex2Cer(d18:1/24:0), Cer(d18:0/24:1), and Cer(d16:1/20:0). In this Pre Tx group, the α-aminobutyric acid was found noticeably diminished (**Figure 5B**).

Levothyroxine therapy (Post Tx group) induced a marked shift in the metabolic profile. Metabolites that were elevated before treatment (Pre Tx group) showed significant reductions post-treatment, reflecting a trend toward normalization of the underlying metabolic pathways. Interestingly, α-aminobutyric acid, which was the only metabolite decreased in the Pre Tx group, exhibited increased levels following hormone replacement (**Figure 5B**).

### 3.4 Biochemical characteristics of the nine metabolites found

In **Table 2**, the biochemical origin, the metabolic pathways and the possible role in thyroid dysfunction of the metabolites that showed statistically significant differences between the studied groups, are summarized.

**Table 2:** Biochemical origins and metabolic pathways of metabolites that differed between the studied congenital hypothyroidism groups.

# HMDB code	Metabolite name, (abbreviation), and chemical group	Metabolic pathway	Possible role in thyroid dysfunction
0000149	Ethanolamine (ETn) Biogenic amine (amino alcohol)	• Precursor for the biosynthesis of two primary phospholipid classes, phosphatidylcholine (PC) and phosphatidylethanolamine (PE) (23, 58). • Essential for PE synthesis via the CDP-ethanolamine (Kennedy pathway) (21, 22, 24).	The thyroid hormone regulates membrane lipid remodeling and mitochondrial energetics; thus, altered ETn/PE metabolism may reflect disrupted mitochondrial function and impaired phospholipid homeostasis in hypothyroidism (6).
0000452	α-aminobutyric acid (AABA). Small neutral α-Amino acid	• Non-proteogenic amino acid derived from sulfur amino acid metabolism, linked to the trans-sulfuration-glutathione axis and produced from methionine and cystathionine turnover, with additional contribution from dietary intake (27).	Because hypothyroidism increases oxidative stress and reduces antioxidant capacity, low or dysregulated AABA may indicate insufficient glutathione regeneration in thyroid hormone deficiency.
0000699	1-Methylnicotinamide (1MNA). Organic compounds known as nicotinamides.	• Produced by nicotinamide N-methyltransferase (NNMT) via S-adenosyl-methionine-dependent methylation of nicotinamide, linking NAD+ metabolism to one-carbon/methylation pathways (59).	Thyroid deficiency alters mitochondrial NAD+ flux and methylation balance. Shifts in 1MNA may represent compensatory vascular signaling or perturbed NAD+/methyl metabolism in thyroid dysfunction.
0000897	7-methylguanine (m7G). Organic compounds known as hypoxanthines	• m7G’s pathways involve enzymatic addition (RNA) or alkylation (DNA), crucial regulatory roles (RNA), and specific removal mechanisms (DNA repair), making it vital for cellular function m7G is one of the most common RNA base modifications in post-transcriptional regulation • It is a degradation product of nucleic acids, and it may be considered an indicator of whole-body RNA turnover. • It inhibits DNA repair enzyme poli(ADP-ribose) polymerase 1 (PARP-1) (32, 60, 61).	Thyroid hormones influence RNA turnover, DNA repair capacity, and renal hemodynamics. Thus, abnormal m7G levels may reflect reduced nucleic acid turnover, altered renal clearance, or impaired DNA-repair signaling in hypothyroidism.
0004880	Hex3Cer (d18:1/18:0) Trihexosylceramide(d18:1/18:0) Glycosphingolipid	• Involved in the construction of complex glycosphingolipids (globotriaosylceramide or Gb3 • Gb3 is generated along the glycosphingolipid biosynthetic axis by sequential addition of glucose and two galactoses to ceramide. It is catabolized by lysosomal alfa.galactosidase A. • Tightly connected to the *de novo* ceramide pathway and the ceramide-sphingosine-sphingosine-1-phosphate axis (38, 62).	Autoimmune thyroiditis and thyroid eye disease are characterized by sphingolipid-rich raft remodeling and acid-sphingomyelinase/ceramide pathway activation in T cells and fibroblasts, suggesting that Gb3 may modulate receptor clustering, antigen presentation, and local inflammatory signaling in Graves disease and Hashimoto´s thyroiditis (39, 41).
0011595	Hex2Cerd(d18:1/24:0) LacCer A very long chain lactosylceramide containing a d18:1 sphingoid base and C24:0 acyl chain.	• LacCer occupies a central node in glycosphingolipid metabolism, being synthesized from glucosylceramide and serving as a precursor for higher glycosphingolipids (globosides and gangliosides). Its catabolism via exoglycosidases and ceramidases feed back into the ceramide. sphingosine-sphingosine-1-phosphate network (38, 62).	Thyroid dysfunction, particularly hypothyroidism and subclinical hypothyroidism, is linked to NAFLD, dyslipidemia, and endothelial dysfunction. LacCer (C24:0) may act as a mediator of oxidative stress, vascular inflammation, and immune activation complications of hypo- and hyperthyroidism (11, 41, 47, 48).
0011768	Cer(d18:0/24:0) Ceramide, C24:0 dhCer A very long-chain dihydroceramide sphingolipids	• C24:0 dhCer is generated by ceramide synthase 2, during *de novo* synthesis in the endoplasmic reticulum ad is converted by dihydroceramide desaturase into C24:0 ceramide (63).	Cer(d18:0/24:0) could reflect thyroid hormone-dependent remodeling of *de novo* sphingolipid flux and may contribute to cardiometabolic complications and altered stress responses in hypo or hyperthyroidism (11, 55).
0011769	Cer(d18:0/24:1) C24:1 dihydroceramide Ceramide Dihydroceramide. A very long-chain dCer subset	• Like other dhCer species, Cer(d18:0/24:1) is formed during *de novo* sphingolipid synthesis and then enzymatically converted into C24:1 ceramide. • Its levels are influenced by substrate availability (palmitoyl-CoA and very-long-chain acyl-CoAs) (35, 62).	To date, to our knowledge, its role in thyroid dysfunction is unknown.
0341517	Cer(d16:1/20:0) Ceramide Sphingolipid	• Component of the sphingolipid metabolism pathway. • Atypical ceramide containing a shorter d16:1 sphingoid base and a saturated C20:0 acyl chain • Present at low abundance in extracellular vesicles and specific epithelial and salivary lipidomes (64). • Generated via alternative serine palmitoyltransferase and ceramide synthase activity Incorporated into the ceramide networks fur further metabolism and intracellular transport (62).	Cer(d16:1/20:0) is consistently associated with NAFLD, increased hepatic fat, impaired fasting glucose, and higher diabetes risk, and is part of ceramide signatures linked to dysglycemia (Gadgil, 2021; 2022). Hypothyroidism, including congenital forms, is associated with NAFLD, dyslipidemia, and altered long-chain acylcarnitines indicative of impaired β-oxidation, as well as perturbations in ceramides and sphingomyelins (8, 11, 52). Overall, Cer(d16:1/20:0) is a candidate biomarker of thyroid-related hepatic steatosis and systemic lipotoxicity.

### 3.5 Inflammation

**Figure 6:**
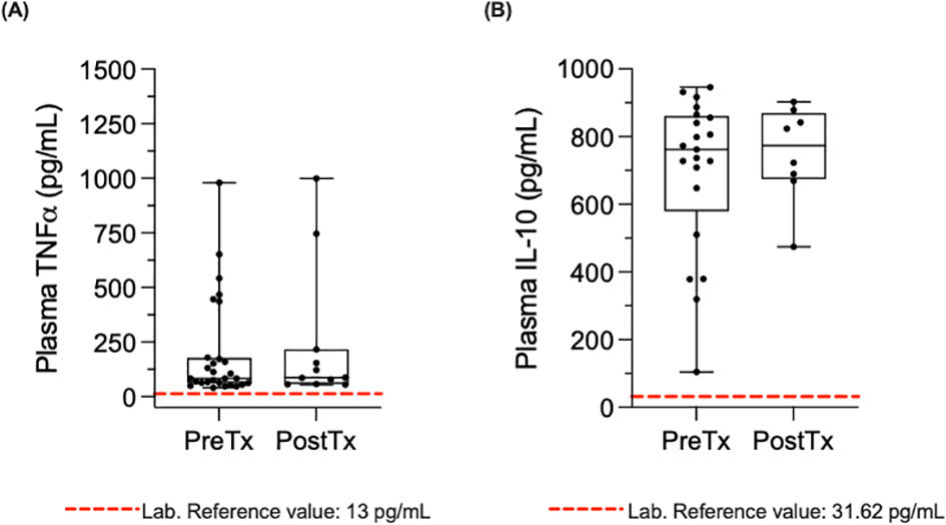
Box and whiskers plot of plasma TNFα **(A)** and IL-10 **(B)** concentrations in patients with congenital hypothyroidism before and after treatment with levothyroxine. No statistical differences were observed between the studied groups; however, cytokine levels remained above laboratory reference ranges in both groups, suggesting persistent inflammation despite treatment.

The inflammatory state of CH patients was investigated before and after the treatment with levothyroxine. Before the treatment started, the median of TNFα concentration was 83.64 pg/mL (**Figure 6A**). This was similar to the blood concentration at treatment initiation, with a median of 87.17 pg/mL. For IL-10, the median concentration before treatment was 761.8 pg/mL, and after treatment it was 773.5 pg/mL (**Figure 6B**). There was no statistical difference in the concentration of both cytokines before and after the treatment onset, however it is worth nothing that these concentrations were notably higher than the reference laboratory values.

## 4 Discussion

This study provides a comprehensive metabolomic characterization of patients with CH in two clinically relevant states: the untreated condition and after restoration of euthyroidism. The main finding was the identification of nine metabolites that significantly differentiate the Pre Tx and Post Tx states. Interestingly, five of these metabolites belonged to the ceramide biochemical group, indicating that lipid-related metabolic pathways, particularly those involving sphingolipids, are substantially disrupted in CH and modulated by thyroid hormone replacement. These results also provide insight into the broad inflammatory and metabolic disturbances present in untreated CH, extending current understanding beyond conventional hormonal assessments.

Among all identified metabolites, ethanolamine showed the most pronounced difference between the two studied groups (Pre Tx and Post Tx), with higher concentrations in the untreated state (**Figure 5A**). Ethanolamine plays a fundamental biological role as precursor (polar head group) of phosphatidylethanolamine (PE), a major phospholipid component of cellular and mitochondrial membranes. Through PE, ethanolamine is essential for membrane biogenesis and structural integrity, mitochondrial oxidative phosphorylation, and the proper folding and function of membrane-associated proteins. It is also required for autophagy initiation and for the biosynthesis of glycosylphosphatidylinositol anchors, which are critical for the membrane localization of numerous proteins. In addition, ethanolamine supports cell proliferation and tissue regeneration and confers cytoprotective effects under metabolic and toxic stress, underscoring its central role in cellular homeostasis and survival (21–25). Until the best of our knowledge, ethanolamine has not previously been associated with thyroid disease. We therefore hypothesize that altered ethanolamine/PE in untreated CH may reflect mitochondrial dysfunction and impaired phospholipid homeostasis secondary to thyroid hormone deficiency.

A distinct pattern was observed for α-aminobutyric acid (AABA), which was reduced in untreated CH patients and increased following restoration of euthyroidism, making it the only metabolite that exhibits this directional change (**Figure 5B**). To our knowledge, AABA has not been reported as an altered metabolite in thyroid dysfunction. Experimental evidence suggests that pro-inflammatory metabolic states are associated with decreased AABA levels, whereas AABA supplementation suppresses glycolysis and promotes oxidative phosphorylation, consequently limiting inflammatory macrophage activation (26). Additionally, AABA has been linked to redox homeostasis through pathways that support glutathione synthesis and mitochondrial function, including AMPK-dependent increases in intracellular glutathione (27). Taken together, these observations suggest that reduced AABA levels in untreated CH patients may reflect impaired anti-inflammatory and redox-adaptative metabolic responses associated with thyroid hormone deficiency. Thus, restoration of euthyroidism could reestablish these protective pathways. Our findings support AABA as a potential component of the metabolomic signature distinguishing untreated CH from the euthyroid state.

Compared with the Post Tx state, 1-methylnicotinamide (1-MNA), was found elevated in the Pre Tx state (**Figure 5C**). The anti-inflammatory and vasoprotective effects of 1-MNA have been attributed to its ability to enhance endothelial nitric oxide synthesis and regulate redox-sensitive signaling pathways, as extensively demonstrated in experimental models (28, 29). Clinically, elevated circulating 1-MNA levels have been associated with adverse cardiovascular outcomes, including left ventricular systolic dysfunction, suggesting that increased 1-MNA may reflect compensatory responses to metabolic or vascular stress rather than exclusively protective signaling (30). In thyroid-related conditions, Du et al. reported reduced 1-MNA levels in orbital adipose tissue from patients with thyroid eye disease; however, this autoimmune disorder may occur independently of the thyroid status (31). Contrary to Du’s findings, our study showed that 1-MNA was elevated in CH untreated patients (**Figure 4C**).

7-methylguanine (m7G) was found elevated in the Pre Tx state (**Figure 5D**). The biological activity of m7G as a PARP-1 inhibitor supports its role in DNA damage responses and in regulating cellular stress pathways (32). More recently, Peng et al. identified m7G as an endogenous biomarker of the glomerular filtration rate (GFR) (33). To our knowledge, no published data have linked m7G to CH. One possible explanation for its altered abundance in untreated CH patients is that it reflects impaired DNA repair signaling in the context of thyroid hormone deficiency (**Figure 4D**).

All remaining metabolites identified as significantly different in this study clustered within the Cer and glycosphingolipid pathways, underscoring a consistent perturbation of sphingolipid metabolism in patients with untreated CH (**Figures 5E–I**). This observation aligns with accumulating evidence that ceramides function not only as structural membrane components but also as central regulators of inflammation, metabolic stress, and cardiometabolic risk (34–36). Ceramides have also been implicated in the pathogenesis of type 2 diabetes, contributing to reduced insulin secretion, impaired insulin signaling, and altered glucose transporter activity, among other metabolic disturbances (34, 37).

In the present study, among the identified ceramides, Hex3Cer(d18:1/18:0), corresponding to globotriaosylceramide (Gb3/GL-3), represents a structurally and functionally relevant glycosphingolipid involved in membrane microdomain organization and receptor-dependent signaling. Gb3 plays a recognized role in immune activation, apoptosis, and endothelial function, and its dysregulated expression is associated with endothelial dysfunction and inflammatory diseases (38–40). Notably, alterations in sphingolipid and cholesterol-enriched membrane microdomains, known as lipid rafts, as well as activation of the acid sphingomyelinase-ceramide pathway, have been described in autoimmune thyroid disorders, including Graves’ disease and Hashimoto thyroiditis, suggesting that Gb3 may influence receptor clustering, antigen presentation, and inflammatory signaling (39, 41).

Lactosylceramide species, represented in this study by Hex2Cer(d18:1/24:0) (**Figure 5F**), are well-established mediators of vascular inflammation and immune activation. Lactosylceramide regulates chemotaxis, phagocytosis, and reactive oxygen species (ROS) generation while activating endothelial NADPH oxidase and cyclooxygenase-2, thereby amplifying pro-inflammatory and pro-atherogenic signaling pathways (42, 43). Human metabolomic studies have linked lactosylceramides to systemic inflammation, impaired lung function, NAFLD, dysglycemia, and increased cardiometabolic risk (44–46). Given the strong association between thyroid dysfunction, especially hypothyroidism and subclinical hypothyroidism, NAFLD, dyslipidemia, and endothelial dysfunction, lactosylceramide (C24:0) may represent a mechanistic link between altered thyroid hormone signaling, oxidative stress, vascular inflammation, and immune activation (11, 41, 47, 48).

Dihydroceramide species, including Cer(d18:0/24:0), are intermediates of *de novo* sphingolipid synthesis regulated by dihydroceramide desaturase (DEGS1). The accumulation of these metabolites may reflect altered sphingolipid flux. Several studies have demonstrated that dihydroceramides modulate the balance between autophagy and apoptosis independently of classical apoptotic pathways (35, 49–52). Very-long-chain ceramides, particularly C24 species, have been implicated in fibrosis, chemotherapy-induced cytotoxicity, cardiometabolic risk, and vascular injury (36, 53). Experimental and clinical data indicate that C24:1 ceramide promotes cellular senescence, mitochondrial dysfunction, ROS generation, and DNA damage, positioning the dihydroceramide/ceramide axis as a critical regulator of cellular stress responses (35, 54). In thyroid disease, these alterations may reflect thyroid hormone-dependent remodeling of *de novo* sphingolipid synthesis, contributing to cardiometabolic complications and impaired cellular stress adaptation (11, 55).

The identification of Cer(d16:1/20:0) further reinforces the link between ceramide dysregulation, hepatic steatosis, and systemic metabolic risk. Human lipidomic studies consistently associate this ceramide with NAFLD, increased hepatic fat content, impaired fasting glucose, and an elevated risk of diabetes (44, 45). Thyroid dysfunction, including congenital and acquired hypothyroidism, is strongly associated with NAFLD, disrupted lipid metabolism, and altered fatty acid β-oxidation, and has also been associated with disturbances in acylcarnitine and sphingolipid profiles (8, 52). Accordingly, Cer(d16:1/20:0) emerges as a candidate biomarker capturing the intersection of thyroid-related hepatic steatosis, lipotoxicity, and low-grade inflammation (8, 11, 45).

From a clinical and nutritional perspective, all patients demonstrated measurable improvement following the initiation of levothyroxine therapy and subsequent restoration of euthyroidism (**Figure 2**; **Table 1**). This clinical recovery coincided with post-Tx metabolomic profiles, suggestive of a shift toward normalization. Nutritional status improved in most patients, evidenced by a progressive trend toward weight normalization, although catch-up linear growth is expected to require a longer time course.

Our findings about alterations in lipid metabolism are concordant with those from the recent study by Guo et al. (6), especially phosphatidylethanolamine, which is an important factor in building membrane structure.

Notably, it should be emphasized that, although no significant differences in TNFα and IL-10 concentrations were observed between the two studied groups, both concentrations were markedly above the laboratory reference values, suggesting the presence of persistent inflammatory state that was not modified in the first three months of treatment. Further longitudinal studies are needed to evaluate the achievement of a regulated state in terms of inflammation in these patients, and to study the mechanisms that underlie this phenomenon.

Collectively, the ceramide-centric metabolic signature and the inflammation biomarkers identified in this study support the concept that altered sphingolipid metabolism represents a convergent pathway linking thyroid dysfunction with immune activation and oxidative stress. These findings highlight the potential utility of ceramides as biomarkers of CH and as possible therapeutic targets.

Overall, our results underscore the profound impact of thyroid hormone replacement on systemic metabolism in patients with CH. The observed metabolic alterations suggest disturbances in amino acid metabolism, energy homeostasis, and potentially gut microbial co-metabolite production, consistent with the metabolic dysregulation associated with hypothyroidism. Importantly, these findings provide insight into the systemic effects of hypothyroidism beyond circulating thyroid hormone levels alone. Some authors have proved that several antioxidant nutrients, such as zinc, selenium, magnesium, and vitamin A, may play an essential role in maintaining normal thyroid function (56, 57). Randomized clinical trials assessing adjunctive therapies, such as antioxidants, vitamin supplementation, or anti-inflammatory agents, are needed to clarify whether targeting these pathways yields additional clinical benefit. Overall, our results indicate that further research is warranted and point to the potential value of complementary therapeutic strategies in CH.

This study has limitations, primarily related to the small sample size. Future research should include longitudinal assessments of inflammatory and oxidative stress markers in patients with CH to better characterize long-term changes. Expanded metabolomic analyses are also warranted to explore associations with neurodevelopmental outcomes, growth, and cardiovascular risk. Besides the small sample size, the lack of stratification by specific etiology (e.g., thyroid dysgenesis versus dishormonogenesis) or sex also represent limitations of this study. However, the longitudinal prospective design, where each patient served as their own internal control, significantly enhanced the statistical power to detect intra-individual metabolic shifts while minimizing inter-individual biological noise. Future multi-center studies with larger cohorts are warranted to validate these metabolic signatures across different clinical phenotypes and to further explore the long-term pharmacometabolic impact of LT4 therapy.

### 4.1 Conclusion

In conclusion, CH is associated with systemic metabolic disturbances that extend beyond thyroid hormone deficiency. This paired metabolomic analysis identified a distinct metabolic signature that distinguished the untreated and euthyroid states, with prominent involvement of sphingolipid and ceramide pathways linked to inflammation, oxidative stress, and metabolic regulation.

Restoration of euthyroidism through levothyroxine therapy was accompanied by broad metabolic normalization and consistent clinical and nutritional improvement in all patients, underscoring the central role of thyroid hormone replacement in systemic recovery. Nevertheless, the metabolic alterations observed in the untreated state suggest that thyroid hormone deficiency exerts effects not fully captured by routine biochemical assessment.

These findings support the potential utility of sphingolipid-related metabolites as biomarkers of disease activity and therapeutic response in CH and highlight the need for further studies to explore adjunctive therapeutic strategies targeting residual inflammatory and oxidative pathways.

## Data Availability

The raw data supporting the conclusions of this article will be made available by the authors, without undue reservation.
